# Thermal, Mechanical and Electrical Properties of Carbon Fiber Fabric and Graphene Reinforced Segmented Polyurethane Composites

**DOI:** 10.3390/nano11051289

**Published:** 2021-05-13

**Authors:** Zhe Shi, Cong Zhang, Xin-Gang Chen, Ang Li, Yang-Fei Zhang

**Affiliations:** School of Materials Science and Engineering, College of Engineering, Peking University, Beijing 100871, China; shizhe@pku.edu.cn (Z.S.); 1601214778@pku.edu.cn (C.Z.); chenxingang@pku.edu.cn (X.-G.C.); li_ang@stu.pku.edu.cn (A.L.)

**Keywords:** carbon fiber fabric, graphene, segmented polyurethane, composites, thermal properties, mechanical properties, electrical properties, thermal interface material

## Abstract

Thermal conductive materials with reliable and high performances such as thermal interface materials are crucial for rapid heat transferring in thermal management. In this work, carbon fiber fabric and graphene reinforced segmented polyurethane composites (CFF-G/SPU) were proposed and prepared to obtain superior thermal, mechanical and electrical properties using the hot-pressing method. The composites exhibit excellent tensile strength and can withstand a tensile force of at least 350 N without breaking. The results show that, comparing with the SPU material, the thermal conductivity is increased by 28% for the CFF-G/SPU composite, while the in-plane electrical conductivity is increased by 8 orders of magnitude to 175 S·m^−1^. The application of CFF-G/SPU composite as a winding thermal interface material with electric-driven self-heating effect presents good performances of fluidity and interface wettability. The composite has great advantages in phase transition and filling the interfacial gap in the short time of few seconds under the condition of electrical field, with the interface temperature difference between two layers significantly reduced.

## 1. Introduction

Thermal management has become a serious problem with the development of modern industry and technology, where thermal conductive materials with reliable and high performances such as thermal interface material is crucial for rapid heat transferring [1]. Segmented polyurethane (SPU) has been rapidly developed in recent years and widely used as adhesives, coatings, biomedical materials and thermal energy storage materials, due to the various design of molecular structure and wide range of adjustable performances [2,3,4]. SPU is usually polymerized by polyester or polyether polyol and isocyanate, with excellent properties of elasticity, wear resistance and corrosion resistance and poor properties of electrical and thermal conductivities [5]. Based on these characteristics, SPU can be used as an excellent polymer matrix for multifunctional composites, compounding with reinforcement materials such as carbon and metal materials to obtain superior performances including electrical conductivity, thermal conductivity, tensile strength and sensing properties, etc. [6,7,8].

Carbon materials such as carbon black, carbon nanotube, carbon fiber and graphene have been widely used in multifunctional composite materials with their unique structures and excellent thermal and electrical properties [9,10,11,12,13,14,15]. However, the content of one-dimensional or two-dimensional fillers must be larger than a percolation value to construct an interconnected structure and provide enough conducting paths for electron transport and heat transfer [16,17]. A high content of filler can easily lead to agglomeration and defects, thereby destroying the mechanical properties of composite material. Three-dimensional interconnected carbon material network prepared before synthesis can avoid the limitation of percolation threshold and provide conducting interconnected structure at very low filler content [18]. The thermal conductivity of the three-dimensional structure system is 66% higher than that of the random dispersion system [19], while the electrical conductivity can be improved by at least 5 orders of magnitude at graphene contents above percolation [20]. However, the carbon content of carbon black, carbon nanotube, carbon fiber and graphene reinforced composites with three-dimensional structures is limited by the preparation methods such as freeze drying, self-assembly and vapor deposition. The thermal and electrical conducting properties is positive correlated with the filler content, so it is difficult to further increase the carbon content and enhance the thermal and electrical conduction effects, and it is also difficult to reduce the thickness to the scale of practical application. Therefore, it is necessary to find a thinner network structure to increase the carbon content and reduce the thickness of the composites while maintaining their thermal, mechanical and electrical performances.

Carbon fiber fabric with ultra-high tensile toughness has been widely used in the mechanical reinforcement of polymer matrix composites [21,22,23,24]. In recent years, carbon fiber fabric has received more and more attention in thermal and electrical conducting polymer composites [25,26], due to the advantages of excellent thermal and electrical conductivities and in-plane interconnected structure. Carbon fiber fabric polymer composites are mainly prepared by the hot-pressing method due to large area of continues structure and micron thickness of carbon fiber fabric. Carbon fiber fabric is widely used in shell materials for mechanical support, electromagnetic shielding, and thermal conduction, because of its high specific strength, high continuity and excellent thermal and electrical conductivities [27,28,29,30]. The thermal conductivity of carbon fiber fabric reinforced polymer composites can be increased by 10 times, while the electrical conductivity can be improved by 10 orders of magnitude [31]. Using carbon fiber fabric as a reinforcement in place of three-dimensional carbon network can effectively improve the electrical and thermal properties of the composites and further reduce the thickness.

In this work, segmented polyurethane (SPU) with solid–solid phase change properties was synthesized. Carbon fiber fabric-graphene/polyurethane composite (CFF-G/SPU) was prepared by the hot-pressing method, as well as the graphene/polyurethane composite (G/SPU) and carbon fiber fabric/polyurethane (CFF/SPU). The thermodynamic properties, electrical conductivity, thermal conductivity and tensile properties are investigated and discussed according to the microscopic morphology. The application of CFF-G/SPU composite as a self-heating phase change thermal interface material is also conducted by experiments of infrared imaging under electrical field and winding on heating tubes.

## 2. Materials and Methods

### 2.1. Materials

The graphene nanosheets was supplied by Ningbo Morsh technology Co. Ltd. (Ningbo, China), with purity > 97.5%, thickness of 1 to 2 nm, diameter of 5 to 15 μm and specific surface area of 150 m^2^·g^−1^, as reported by supplier. The carbon fiber fabric (plain-3K-2) was obtained from 3M China Co. Ltd. (Shanghai, China), with carbon fiber diameter of 5 to 8 μm, tensile strength > 3300 MPa and tensile modulus > 220 MPa, as reported by supplier. Polyethylene glycol(PEG) 6000, 4,4′-diphenylmethane diisocyanate(MDI) and 1,4-butanediol(BDO) were provided by Shanghai Aladdin Co. Ltd., (Shanghai, China). Other chemicals were purchased from Sinopharm Chemical Reagent Co. Ltd. (Shanghai, China).

### 2.2. Synthesis of Segmented Polyurethane (SPU)

The Segmented Polyurethane (SPU) was prepared by in situ polymerization method. PEG was dried in vacuum (about 100 kPa), and heated to 100 °C for 2–3 h to remove the moisture. Then, MDI and BDO were added to PEG according to the stoichiometric ratio, and then viscous mixture was fully stirred. Finally, the mixture was heated in a dry environment of 75 °C for 24 h. The isocyanate group (-NCO) in MDI reacted with the hydroxyl group (-OH) in PEG and with BDO to form a carbamate group (-NH-COO-), then the SPU material was obtained.

### 2.3. Preparation of Composites

Graphene/segmented polyurethane composite (G/SPU) was prepared by solution blending method. Then, 2.97 g SPU was added into 50 mL N,N-dimethylformamide (DMF), then heated to 60 °C and stirred continuously to make SPU completely dissolved. Next, 0.03 g graphene nanosheets were added and stirred for 20 min. After the mixed solution cooled, ultrasonic treatment was carried out for 20 min to make graphene completely mixed and homogenously dispersed. The mixed solution was poured into a mold, heated at 70 °C for 24 h to remove DMF solvent, and then G/SPU composite was obtained.

**Figure 1:**
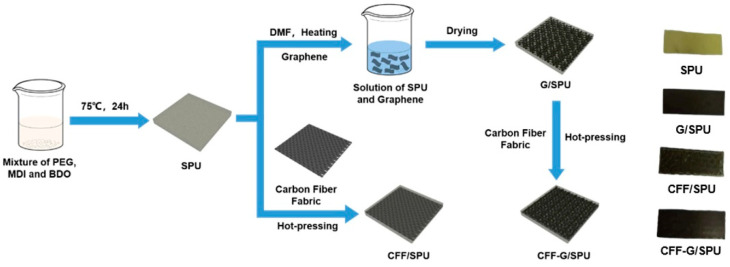
Schematic illustration of preparation method of SPU, G/SPU, CFF/SPU and CFF-G/SPU materials and their photographs.

Carbon fiber fabric/segmented polyurethane (CFF/SPU) composite was prepared by hot-pressing method. SPU was shaped into thin sheets by hot-pressing in advance, then a carbon fiber fabric was placed between two SPU sheet and the compound was hot pressed. Carbon fiber fabric and graphene reinforced segmented polyurethane composite (CFF-G/SPU) was prepared by a similar method with CFF/SPU, using G/SPU as outer layer instead of SPU. The samples of SPU, G/SPU, CFF/SPU and CFF-G/SPU were cut into strips with size of 1 cm × 3 cm and exhibited in Figure 1. The SPU matrix obtained is transparent and light yellow, while the colors of G/SPU, CFF-SPU and G-CFF/SPU samples are black and opaque, with textures of carbon fiber fabric obviously observed through the surface of samples.

### 2.4. Characterization

The microstructure of composites was observed by a scanning electron microscope (SEM) (S4800, Hitachi Co., Tokyo, Japan) with accelerating voltage of 5 kV and current of 10 μA. The observation surfaces of SPU and G/SPU samples were obtained by Freezing brittle fracture in liquid nitrogen. Due to the high toughness of carbon fiber fabric, the observation surfaces of CFF/SPU and CFF-G/SPU samples were obtained by mechanical cutting.

The phase transition temperature, enthalpy and specific heat capacity were measured by differential scanning calorimetry (DSC) (DSC Q2000, TA Co., New Castle, DE, USA) with a temperature range from −20 °C to 90 °C and under a rate of 10 °C·min^−1^ for temperature rise. Specific heat capacities were calculated from sapphire line and baseline. The temperature filed was measured by an infrared camera (T420, FLIR Co., Goleta, CA, USA).

The mechanical properties were measured by a universal material testing machine (CMT6103, SANS, Shanghai, China) with loading rate of 5 mm/s. The densities were measured by an automatic density analyzer (XS204, Mettler-Toledo AG, Greifensee, Switzerland).

The electrical conductivities of composites were measured by a digital source meter (Keithley 2400, Keithley Co., Cleveland, OH, USA) under a stable direct current (DC) voltage of 2.1 V. The thermal conductivities of SPU, G/SPU, CFF/SPU, CFF-G/SPU samples were measured by a self-build test system based on the steady-state plate method. The system has been reported in our previous work [32].

## 3. Results and Discussion

### 3.1. Microstructures

**Figure 2:**
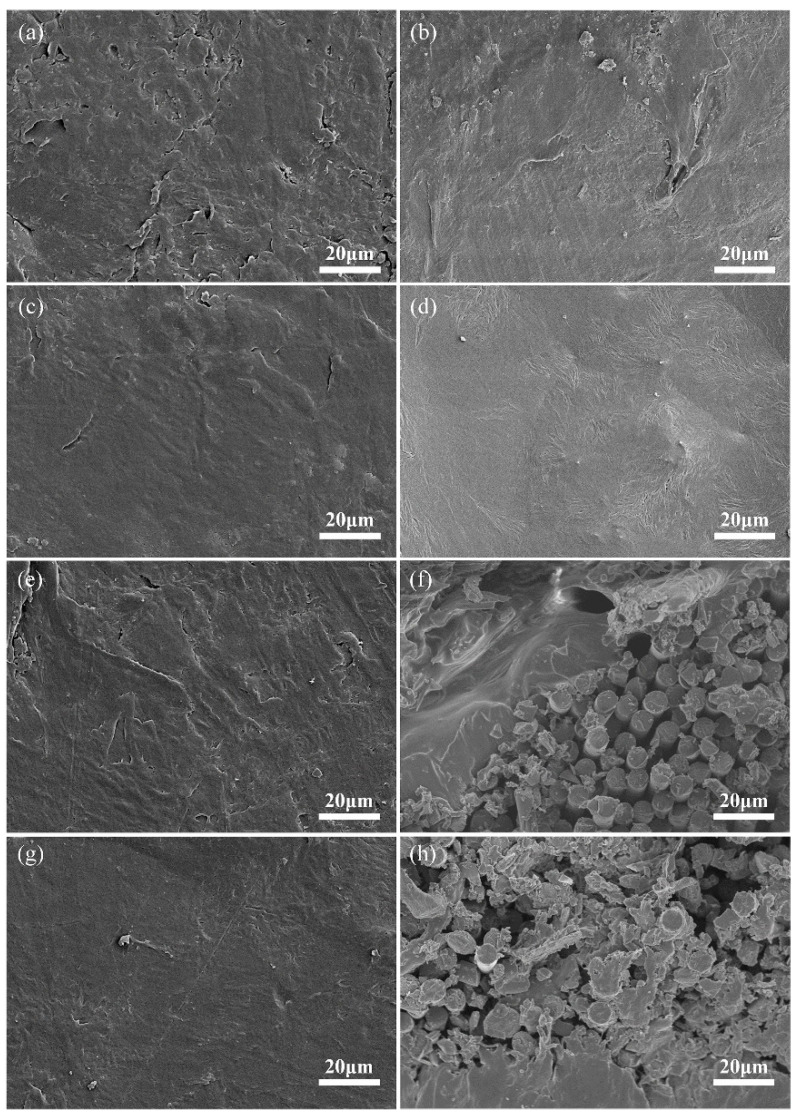
Surface and cross section micrographs of (**a**,**b**) SPU, (**c**,**d**) G/SPU, (**e**,**f**) CFF/SPU and (**g**,**h**) CFF-G/SPU.

Figure 2 shows the microscopic morphology of surface and liquid nitrogen frozen fractured surface of SPU, G/SPU, CFF/SPU and CFF-G/SPU samples. It can be observed from Figure 2b that the fractured surface of SPU matrix is relatively smooth and flat, while the surface of G/SPU composite with graphene sheets presents some wavy wrinkles, as shown in Figure 2d, indicating that graphene sheets are homogenously dispersed in SPU matrix. Figure 2f,h show the microstructures of composite materials with carbon fiber fabric. It can be measured from the figure that the fiber diameter of carbon fiber fabric is about 7 μm. The matrix is successfully melted and penetrated into the space between carbon fibers during the process of hot-pressing, while the adding of graphene has little effect on the structure. It is beneficial for good structural integrity of composite and strong interface between SPU matrix and carbon fiber fabric.

### 3.2. Thermal Properties

**Figure 3:**
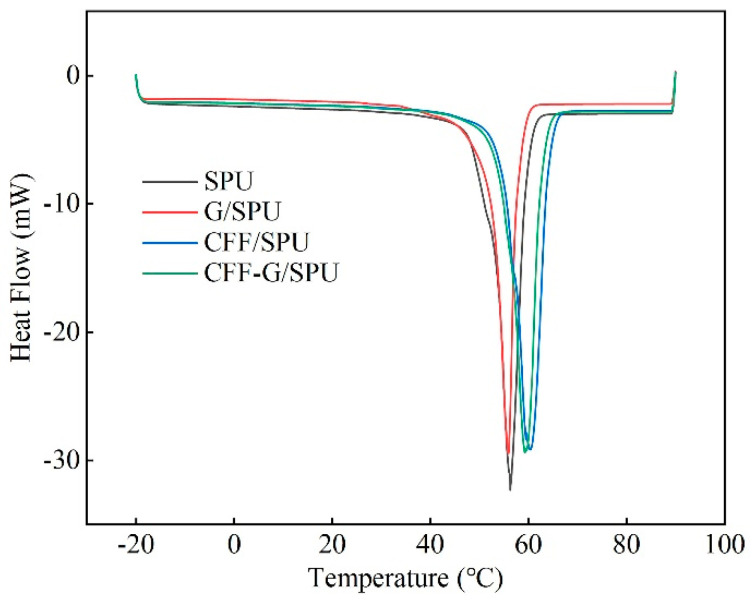
DSC curves of SPU, G/SPU, CFF/SPU and CFF-G/SPU.

The thermodynamic properties of samples were measured by differential scanning calorimetry (DSC), as shown in Figure 3. By comparing the phase transition temperatures of SPU and CFF/SPU with those of G/SPU and G-CFF/SPU, it is found that phase transition temperature of composites with graphene has a slight decrease, from 56.3 °C to 55.9 °C, and from 60.4 °C to 59.2 °C, respectively. While the heat of fusion is 127.0 J·g^−1^, 125.8 J·g^−1^, 113.3 J·g^−1^ and 102.9 J·g^−1^ for SPU, G/SPU, CFF/SPU and G-CFF/SPU, respectively. The slightly decrease in the fusion heat of composites indicates a decrease in crystallinity with the adding of graphene and carbon fiber fabric. This is due to that semi-crystalline structure of SPU, composing by soft segments of PEG and hard segments of MDI, is destroyed by the adding of graphene, which makes the phase transition easier. Compared with graphene nanosheets, carbon fiber fabric woven composed by long carbon fibers is a continuous and stable structure, which restricts the movement of the molecular chain segments and increases the phase transition temperatures by 4.1 °C and 3.3 °C, respectively. The specific heat capacity of the SPU is 1.78 J·g^−1^·K^−1^, and that of the G/SPU is 1.61 J·g^−1^·K^−1^. It is due to the low specific heat capacity of graphene (0.7 J·g^−1^·K^−1^) [33], 1% mass fraction reduces the specific heat by 9.6%. The specific heat capacity of CFF/SPU and CFF-G/SPU are both decreased to about 1.36 J·g^−1^·K^−1^.

**Figure 4:**
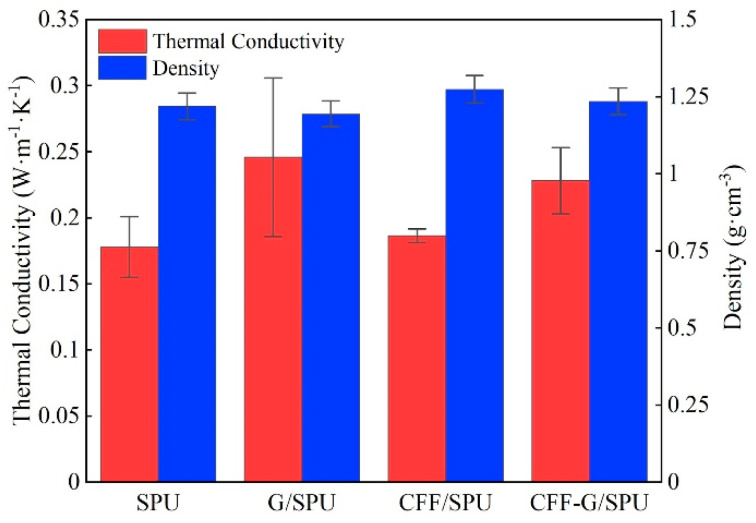
Thermal conductivities and densities of SPU, G/SPU, CFF/SPU and CFF-G/SPU.

The thermal conductivities of samples were measured by steady-state plate method, while the values were the average of three measurements. The thermal conductivity of SPU matrix is 0.178 W·m^−1^·K^−1^, while that of G/SPU is 0.246 W·m^−1^·K^−1^, as shown in Figure 4. It is found that adding 1 wt.% graphene can improve the thermal conductivity of composite by about 38%. However, the adding of carbon fiber fabric with high in-plane thermal conductivity and content of 24 wt.% has little influence on the thermal conductivity of CFF/SPU composites. This is due to the three-layer structure of CFF/SPU composites requiring that the heat must pass through two SPU-CFF interfaces and result in a low conductive efficiency, when the heat flow direction is perpendicular to the fiber direction. The addition of graphene can significantly improve the through-plane thermal conductivity of CFF/SPU and achieve a higher thermal conductivity of CFF-G/SPU composite. Because of the higher density and content of carbon fiber fabric, the composites of CFF/SPU and CFF-G/SPU have slightly higher densities.

### 3.3. Mechanical Properties

**Figure 5:**
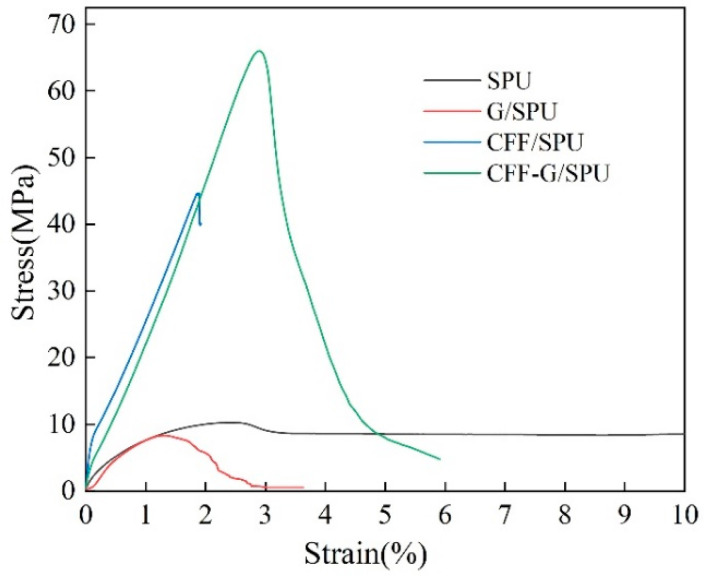
Stress–strain curses of SPU, G/SPU, CFF/SPU and CFF-G/SPU.

The mechanical properties of four materials were characterized by tensile testing, and tensile moduli were calculated from elastic segments, as shown in Table 1. After adding of graphene and carbon fiber fabric reinforcement fillers, the CFF-G/SPU composites represent the best mechanical properties, as shown in Figure 5. The tensile modulus is increased by 4 times to 2317 MPa, while the tensile strength is increased by 6.4 times to 66.1 MPa. However, the tensile strength of G/SPU composite is decreased by 19.42% to 8.3 MPa, attributed to that the high specific surface area of graphene nanosheets are easy to cause agglomeration and defects. Because of the three-layer structures of CFF/SPU and CFF-G/SPU composites, the samples will slip from the fixtures of testing machine before the broken of carbon fiber fabric, which means the highest point of stress–strain curve is the maximum stress when the sample slips, not the maximum stress when it breaks. Therefore, the theoretically tensile strength of carbon fiber fabric reinforced composite should be larger. It can be explained by the introduction of carbon fiber fabric into SPU matrix provides excellent mechanical support for composite materials and greatly improves the mechanical properties.

### 3.4. Electrical Properties

**Figure 6:**
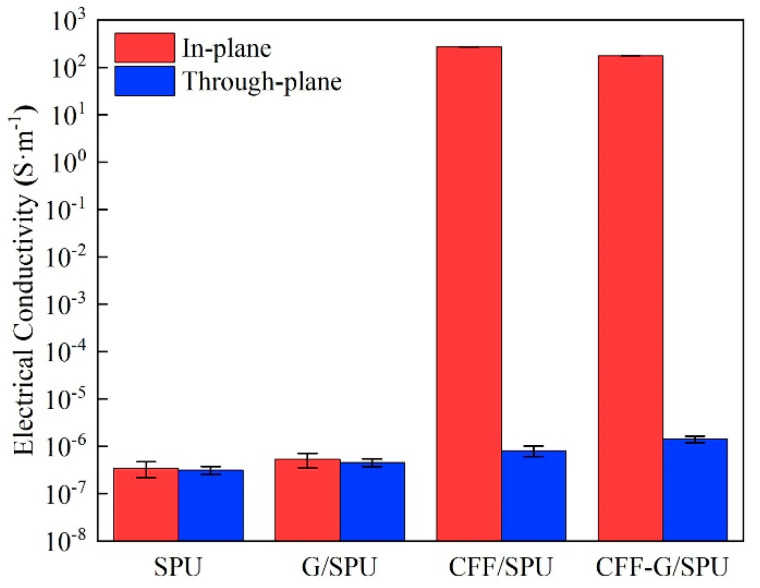
In-plane and through-plane electrical conductivities of SPU, G/SPU, CFF/SPU and CFF-G/SPU.

The in-plane and through-plane electrical conductivities of SPU, G/SPU, CFF/SPU and CFF-G/SPU are shown in Figure 6. The result shows that the in-plane conductivities of CFF/SPU and CFF-G/SPU composites have been significantly improved with an increase of about 8 orders of magnitude. The unique two-dimensional structure of carbon fiber fabric provides fast electronic transmission channels along the fiber direction and enhances the in-plane conductivity of composite. As the content of 1 wt.% graphene is not enough to form an interconnected electrical conducting structure, the in-plane and through-plane conductivities of G/SPU composites are slightly increased due to the high conductivity of randomly dispersed graphene nanosheets. The through-plane conductivities of CFF/SPU and CFF-G/SPU composites increase sequentially in a small range, attributed to that the high conductivity carbon fiber fabric is covered by SPU matrix in the through-plane direction due to the three-layer structure of CFF/SPU and CFF-G/SPU composites.

### 3.5. Electric-Driven Self-Heating Effect and Thermal Interface Material Application

Because of the high thermal conductivity, CFF-G/SPU composite has good potential as a thermal interface material. When the temperature rises to phase transition temperature, the soft segments of SPU matrix become amorphous and fill the interface voids, resulting in good interfacial wettability and low thermal contact resistance. The modulus of SPU after phase change is very small and cannot withstand large pressure, while the carbon fiber fabric with good toughness and compression resistance can greatly improve the modulus of composite and withstands large pressure at high temperature after phase change. In addition, the thermal expansion of carbon fiber fabric is small, which can limit the expansion and contraction of SPU matrix. With the advantages of high electrical conductivity and in-plane thermal conductivity of carbon fiber fabric, CFF-G/SPU composite exhibits electric driven self-heating effect and beneficial to the pre-assembly of thermal interface material between the heating device and the heat sink.

**Figure 7:**
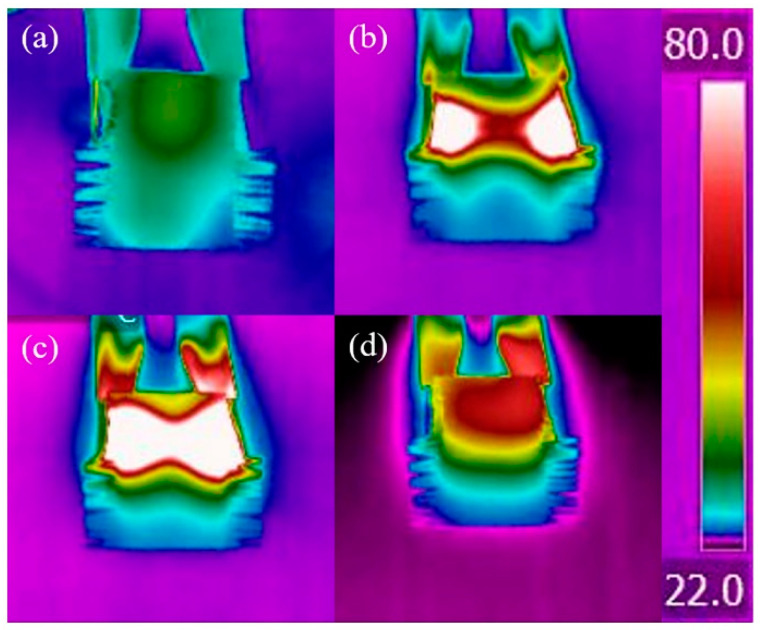
Temperature distribution of CFF-G/SPU composite: (**a**) without electricity; (**b**) under electric field for 10 s; (**c**) power off for 30 s; and (**d**) 60 s.

To demonstrate the electric driven self-heating effect of CFF-G/SPU composite, an electric field with a voltage of 3 V and a power of 5.1 W was applied at both ends of the carbon fiber fabric to heat the composite and raise the temperature above the phase transition temperature, with an infrared camera used to observe the temperature distribution of composite surface, as shown in Figure 7. When the power is turned on, the heat concentration is located at the contact region of alligator clip and carbon fiber fabric, where the resistance is the largest. Due to the extremely high thermal conductivity of carbon fiber, the heat is rapidly transmitted from the outside to the inside along the axial direction. The temperature rapidly increased to over 150 °C under electric field for 10 s, and then the phase transition of composite can be obviously observed, which means the pre-assembly process of thermal interface material can be finished in 10 s by the electric driven self-heating effect. When it reaches 30 s after the power turned off, the temperature distribution becomes homogenous and the temperature of all regions is over 80 °C. At 60 s after the power turned off, the temperature of the composites homogenously decreased to 40 °C, lower than the phase transition temperature.

**Figure 8:**
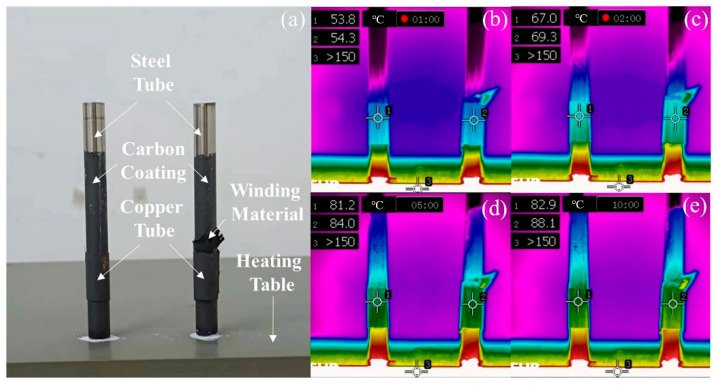
(**a**) Photograph of CFF-G/SPU composite as a winding thermal interface material and temperature distribution after heating for (**b**) 1 min, (**c**) 2 min, (**d**) 5 min and (**e**) 10 min.

Due to the excellent mechanical properties and layer structure, CFF-G/SPU composite is especial suitable as a winding thermal interface material for the connection and heat dissipation of equipment such as cooling sleeve. As carbon fiber fabric can be used as a long continuous reinforcement, CFF-G/SPU composite becomes a natural tape-like winding material with high thermal conductivity and large tensile strength, which is beneficial for the heat dissipation when connecting the inner and outer tubes. In addition, the electrical conductivity in the through-plane direction is extremely low, equivalent to an insulator, so the short circuit can be avoided and the electric driven self-heating method is still available to obtain stronger connection interface between the inner and outer tubes. In this work, a strip of CFF-G/SPU composite with a width of 0.5 cm was used as the winding thermal interface material, while a stainless-steel tube and a copper tube were used as the inner tube and outer tube, respectively. The prepared sleeves were located on a heating table at constant temperature of 180 °C, while carbon powder was prayed on the surface of sleeves for better infrared imaging, as shown in Figure 8. The temperature of cooper cube rises to 54.3 °C, 69.3 °C, 84.0 °C and 88.1 °C at 1 min, 2 min, 5 min, 10 min, respectively, and then remain stable with a temperature difference between sleeves of only 5.2 °C. It proves that the winding thermal interface material of CFF-G/SPU composite enhancing the heat dissipation performance and reducing the temperature difference between the inner and outer tubes.

## 4. Conclusions

In this work, segmented polyurethane was synthesized, and carbon fiber fabric and graphene nanosheets were used as reinforcements to enhance the composite with high thermal, mechanical and electrical properties. Four materials of SPU, G/SPU, CFF/SPU and CFF-G/SPU were successfully prepared and their microscopic morphology, mechanical properties, thermal properties and electrical properties were carefully characterized. The results show that the CFF-G/SPU composite has the largest tensile strength and can withstand a tensile force of at least 350 N without breaking. The thermal stability of CFF/SPU and CFF-G/SPU composites has been improved with the phase transition temperatures increased by 4.1 °C and 2.9 °C, respectively. Compared with SPU materials, the thermal conductivity of G/SPU is increased by 28% to 0.246 W·m^−1^·K^−1^ at a low content of 1 wt.%, with in-plane and through-plane electrical conductivities slightly increased. The tensile strength of G/SPU is increased by 112.97%, while the tensile strength is decreased by 19.42%. The in-plane electrical conductivity of CFF/SPU and CFF-G/SPU are significantly improved by 8 orders of magnitude. The experiments of electric-driven self-heating and application as a tape-like winding thermal interface material proves that CFF-G/SPU composites have great potential in thermal interface material with good interface wettability, high thermal conductivity and large tensile strength.

## Figures and Tables

**Table 1:** Mechanical properties of SPU, G/SPU, CFF/SPU and CFF-G/SPU.

Sample	Thickness (mm)	Tensile Modulus (MPa)	Tensile Strength (MPa)
SPU	0.52	609	10.3
G/SPU	0.30	1297	8.3
CFF/SPU	0.78	2100	44.6
CFF-G/SPU	0.76	2317	66.1

## Data Availability

Not applicable.

## References

[1] Ma J.K., Shang T.Y., Ren L.L., Yao Y.M., Zhang T., Xie J.Q., Zhang B.T., Zeng X.L., Sun R., Xu J.B. (2020). Through-plane assembly of carbon fibers into 3D skeleton achieving enhanced thermal conductivity of a thermal interface material. Chem. Eng. J..

[2] Grenier S., Sandig M., Mequanint K. (2007). Polyurethane biomaterials for fabricating 3D porous scaffolds and supporting vascular cells. J. Biomed. Mater. Res. Part A.

[3] Cai D., Yusoh K., Song M. (2009). The mechanical properties and morphology of a graphite oxide nanoplatelet/polyurethane composite. Nanotechnology.

[4] Xiang C.S., Lu W., Zhu Y., Sun Z.Z., Yan Z., Hwang C.C., Tour J.M. (2012). Carbon nanotube and graphene nanoribbon-coated conductive Kevlar fibers. ACS Appl. Mater. Interfaces.

[5] Loh T.W., Tran P., Das R., Ladani R.B., Orifici A.C. (2020). Thermoplastic polyurethane-cellulose nanocomposite for transparent armour: Characterisation of adhesion and thermal aging. Compos. Commun..

[6] Li W., Pei Z. (2021). Strain-sensing fiber with a core–sheath structure based on carbon black/polyurethane composites for smart textiles. Text. Res. J..

[7] Gedam S.S., Chaudhary A.K., Vijayakumar R.P., Goswami A.K., Bajad G.S., Pal D. (2019). Thermal, mechanical and morphological study of carbon nanotubes-graphene oxide and silver nanoparticles based polyurethane composites. Mater. Res. Express.

[8] Pourmohammadi-Mahunaki M., Haddadi-Asl V., Roghani-Mamaqani H., Koosha M., Yazdi M. (2020). Preparation of polyurethane composites reinforced with halloysite and carbon nanotubes. Polym. Compos..

[9] Geim A.K., Novoselov K.S. (2007). The rise of graphene. Nat. Mater..

[10] Balandin A.A., Ghosh S., Bao W.Z., Calizo I., Teweldebrhan D., Miao F., Lau C.N. (2008). Superior thermal conductivity of single-layer graphene. Nano Lett..

[11] Pan D., Zhang J., Li Z., Wu M. (2010). Hydrothermal route for cutting graphene sheets into blue-luminescent graphene quantum dots. Adv. Mater..

[12] Trache D., Thakur V.K., Boukherroub R. (2020). Cellulose nanocrystals/graphene hybrids-a promising new class of materials for advanced applications. Nanomaterials.

[13] Platnieks O., Gaidukovs S., Neibolts N., Barkane A., Gaidukova G., Thakur V.K. (2020). Poly (butylene succinate) and graphene nanoplatelet–based sustainable functional nanocomposite materials: Structure-properties relationship. Mater. Today Chem..

[14] Sharma B., Thakur S., Trache D., Nezhad H.Y., Thakur V.K. (2020). Microwave-assisted rapid synthesis of reduced graphene oxide-based gum tragacanth hydrogel nanocomposite for heavy metal ions adsorption. Nanomaterials.

[15] Siwal S.S., Zhang Q., Sun C., Thakur V.K. (2020). Graphitic carbon nitride doped copper–manganese alloy as high–performance electrode material in supercapacitor for energy storage. Nanomaterials.

[16] Shante V.K.S., Kirkpatrick S. (1987). An introduction to percolation theory. Phys. Today.

[17] Kolonelou E., Loupou E., Klonos P., Sakellis A.E., Valadorou D., Kyritsis A., Papathanassiou A.N. (2021). Thermal and electrical characterization of poly(vinyl)alcohol)/poly (vinylidene fluoride) blends reinforced with nano-graphene platelets. Polymer.

[18] Pettes M.T., Ji H.X., Ruoff R.S., Shi L. (2012). Thermal transport in three-dimensional foam architectures of few-layer graphene and ultrathin graphite. Nano Lett..

[19] Jia J.J., Sun X.Y., Lin X.Y., Shen X., Mai Y.W., Kim J.K. (2014). Exceptional electrical conductivity and fracture resistance of 3D interconnected graphene foam/epoxy composites. ACS Nano.

[20] Tang L.G., Kardos J.L. (1997). A review of methods for improving the interfacial adhesion between carbon fiber and polymer matrix. Polym. Compos..

[21] Zhang L.B., Li R.Y., Tang B., Wang P. (2016). Solar-thermal conversion and thermal energy storage of graphene foam-based composites. Nanoscale.

[22] Zhu S., Shi R.J., Qu M.C., Zhou J.F., Ye C.H., Zhang L.Y., Cao H.J., Ge D.T., Chen Q.J. (2021). Simultaneously improved mechanical and electromagnetic interference shielding properties of carbon fiber fabrics/epoxy composites via interface engineering. Compos. Sci. Technol..

[23] Rahmani H., Najafi S.H.M., Ashori A. (2014). Mechanical performance of epoxy/carbon fiber laminated composites. J. Reinf. Plast. Compos..

[24] Kim J., Cha J., Chung B., Ryu S., Hong S.H. (2020). Fabrication and mechanical properties of carbon fiber/epoxy nanocomposites containing high loadings of noncovalently functionalized graphene nanoplatelets. Compos. Sci. Technol..

[25] Forintos N., Czigany T. (2019). Multifunctional application of carbon fiber reinforced polymer composites: Electrical properties of the reinforcing carbon fibers—A short review. Compos. Part B Eng..

[26] Fan W., Li J.L., Zheng Y.Y., Liu T.J., Tian X., Sun R.J. (2016). Influence of thermo-oxidative aging on the thermal conductivity of carbon fiber fabric reinforced epoxy composites. Polym. Degrad. Stabil..

[27] Yu S., Park K., Lee J.W., Hong S.M., Park C., Han T.H., Koo C.M. (2017). Enhanced thermal conductivity of epoxy/Cu-plated carbon fiber fabric composites. Macromol. Res..

[28] Chen S., Feng J. (2014). Epoxy laminated composites reinforced with polyethyleneimine functionalized carbon fiber fabric: Mechanical and thermal properties. Compos. Sci. Technol..

[29] Duan N.M., Shi Z.Y., Wang J.L., Wang G.L., Zhang X.Z. (2020). Strong and flexible carbon fiber fabric reinforced thermoplastic polyurethane composites for high-performance EMI shielding applications. Macromol. Mater. Eng..

[30] Abot J.L., Yasmin A., Jacobsen A.J. (2004). In-plane mechanical, thermal and viscoelastic properties of a satin fabric carbon/epoxy composite. Compos. Sci. Technol..

[31] Jang J.U., Park H.C., Lee H.S., Khil M.S., Kim S.Y. (2018). Electrically and thermally conductive carbon fibre fabric reinforced polymer composites based on nanocarbons and an in-situ polymerizable cyclic oligoester. Sci. Rep..

[32] Zhang C., Shi Z., Li A., Zhang Y.F. (2020). Rgo-coated polyurethane foam/segmented polyurethane composites as solid–solid phase change thermal interface material. Polymers.

[33] Li Q.Y., Xia K.L., Zhang J., Zhang Y.Y., Li Q.Y., Takahashi K., Zhang X. (2017). Measurement of specific heat and thermal conductivity of supported and suspended graphene by a comprehensive Raman optothermal method. Nanoscale.

